# Effects of beetroot juice supplementation on maximal oxygen uptake during aerobic exercise: a systematic review and meta-analysis

**DOI:** 10.3389/fnut.2026.1858216

**Published:** 2026-07-14

**Authors:** Gang Qin, Sungmin Kim

**Affiliations:** 1 Major in Sport Science, College of Performing Arts and Sport, Hanyang University, Seoul, Republic of Korea; 2 BK21FOUR, Human-Tech Convergence Program, Hanyang University, Seoul, Republic of Korea; 3 Center for Artificial Intelligence Muscle, Hanyang University, Seoul, Republic of Korea

**Keywords:** aerobic performance, beetroot juice, dietary nitrate, meta-analysis, sports nutrition, VO_2_max, VO_2_peak

## Abstract

**Background:**

Beetroot juice (BRJ) is a dietary source of inorganic nitrate that may increase nitric oxide bioavailability and influence aerobic exercise capacity. Previous reviews have included scattered evidence on maximal or peak oxygen uptake in the context of overall exercise performance. This review assessed the effect of BRJ supplementation on VO₂max/VO₂peak in healthy adults.

**Methods:**

This systematic review and meta-analysis of randomized and crossover placebo-controlled trials was conducted in accordance with PRISMA 2020. A total of six databases were searched from inception to 31 January 2026. To be eligible for inclusion, studies had to involve terms such as healthy adults, BRJ or beetroot-derived nitrate supplementation, an incremental or graded aerobic exercise test, a placebo comparator, and reporting of VO₂max or VO₂peak. Pooled effects were calculated as Hedges’ g under a random-effects model.

**Results:**

Thirty-eight studies involving 703 participants were included in the quantitative synthesis. BRJ produced a small statistically significant improvement in VO₂max/VO₂peak compared with placebo (SMD = 0.24, 95% CI [0.11, 0.37], *p* < 0.001; I^2^ = 48.3%). Effects were larger in recreationally active participants (SMD = 0.38) and were not clearly demonstrated in highly trained athletes (SMD = 0.07, 95% CI [−0.09, 0.23]). Standard nitrate doses of 6.4–12.8 mmol NO₃^−^ and supplementation lasting at least 3 consecutive days showed the most consistent effects. Funnel plot asymmetry suggested possible small-study effects; after trim-and-fill adjustment, the pooled estimate was attenuated but remained statistically significant (SMD = 0.19, 95% CI [0.07, 0.31]).

**Conclusion:**

BRJ supplementation is associated with a small improvement in VO₂max/VO₂peak, particularly in recreationally active adults. The effect may be smaller after adjusting for publication bias, and evidence in highly trained athletes remains limited and uncertain. The overall certainty of evidence was rated as moderate.

**Systematic Review Registration:**

https://www.crd.york.ac.uk/prospero/. PROSPERO registration: CRD420261414721.

## 1 Introduction

Maximal oxygen uptake (VO₂max) is one of the most widely used measures of cardiorespiratory fitness and aerobic capacity (1). In many exercise studies, however, participants do not always meet the criteria needed to confirm a true VO₂max, such as an oxygen uptake plateau or other accepted markers of maximal effort. In such cases, the highest oxygen uptake recorded during the test is more accurately described as VO₂peak. This is significant because the use of verified VO₂max combined with unverified VO₂peak could introduce methodological variation and make it difficult to interpret the pooled results. The use of beetroot juice as a sports nutrition supplement is of interest because of its high inorganic nitrate (NO₃^−^) content. Nitrate is also converted to nitrite and subsequently nitric oxide in the gut after absorption, which plays a role in vascular function, oxygen delivery, and muscle contraction (2). These effects are considered to be biologically plausible and support the expectation that beetroot juice could have an effect on aerobic exercise performance. However, the magnitude of these effects is unlikely to be the same in all populations due to variations in training status, dose, supplementation duration, and exercise testing method. Dietary nitrate or beetroot juice has been reviewed previously in relation to a number of exercise-performance outcomes, such as time to exhaustion, exercise economy, and endurance performance (3). VO₂max/VO₂peak, however, has been reported as a subgroup within larger studies. Furthermore, prior syntheses have not always distinguished between verified VO₂max and VO₂peak data, as this reduced the accuracy of their findings. The present systematic review and meta-analysis only included RCTs investigating the impact of beetroot juice supplementation on VO₂max/VO₂peak during aerobic exercise for this reason. A further aim was to explore whether supplementation duration, nitrate dose, training status, exercise modality, and methodological differences influenced the observed effects.

## 2 Materials and methods

### 2.1 Study design and protocol registration

This systematic review and meta-analysis was conducted in accordance with the Preferred Reporting Items for Systematic Reviews and Meta-Analyses (PRISMA) 2020 guidelines (4). The review protocol was retrospectively registered with the International Prospective Register of Systematic Reviews (PROSPERO; registration number: CRD420261414721) after the initial review process had started. A copy of the registered protocol is provided as Supplementary File 1 to ensure transparency. The research question was formulated according to the PICO framework as follows: (Population - P) healthy adults or trained athletes undergoing aerobic exercise, (Intervention - I) beetroot juice or beetroot-derived nitrate supplementation, (Comparison - C) placebo, and (Outcome - O) maximal or peak oxygen uptake (VO₂max or VO₂peak).

### 2.2 Eligibility criteria

Eligible studies met the following criteria: (i) randomized controlled or randomized crossover design; (ii) BRJ or beetroot-derived dietary nitrate as the experimental intervention; (iii) placebo comparator, preferably nitrate-depleted BRJ; and (iv) reporting of VO₂max or VO₂peak measured during incremental or graded aerobic exercise testing. Studies were excluded if they lacked a placebo comparator, used only non-beetroot nitrate sources, included clinical populations likely to confound aerobic-capacity assessment, combined BRJ with other interventions such that the independent effect could not be isolated, or did not provide sufficient data for effect-size calculation.

### 2.3 Information sources and search strategy

A systematic literature search was conducted in PubMed/MEDLINE, Scopus, Web of Science Core Collection, SPORTDiscus via EBSCOhost, Cochrane CENTRAL, and Google Scholar. All databases were searched from inception to 31 January 2026. Reference lists of included studies and relevant reviews were also searched manually. No date restriction was applied during the search stage; however, only English-language full-text articles were included in the final analysis.

Medical Subject Headings (MeSH) terms and free-text keywords were used to construct the search strategy, with a combination of terms and keywords related to the intervention, population, and outcome. Search terms were combined using the Boolean operators AND and OR. The basic search query that was modified to suit this database was as follows:

*(beetroot juice OR beet root juice OR Beta vulgaris OR dietary nitrate OR inorganic nitrate OR nitrate supplementation) AND (VO₂ max OR VO₂ peak OR maximal oxygen uptake OR maximal oxygen consumption OR aerobic capacity OR cardiorespiratory fitness) AND (exercise OR aerobic exercise OR endurance exercise OR physical performance) AND (randomized controlled trial OR randomised controlled trial OR crossover OR clinical trial)*.

The first search was not limited to any date or language, but only English-language full-text studies were incorporated in the final analysis.

### 2.4 Study selection

**Figure 1:**
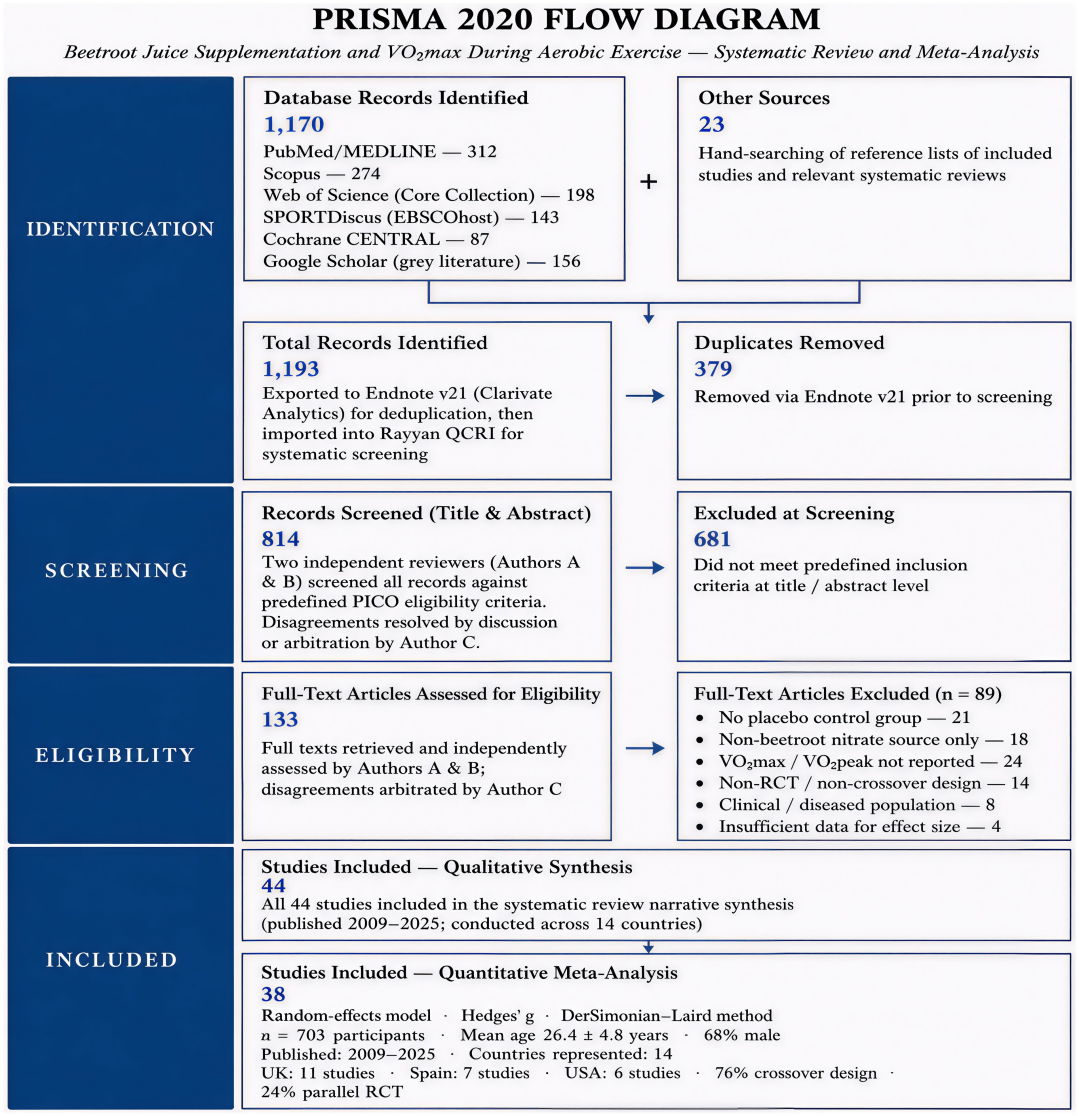
PRISMA 2020 flow diagram of study identification, screening, eligibility assessment, and inclusion. Records were deduplicated using EndNote v21 and screened using Rayyan QCRI.

All search records were exported into EndNote (version 21, Clarivate Analytics) and then imported into the Rayyan QCRI software for systematic deduplication and screening. After deduplication, all records were screened at the title and abstract level by two independent reviewers (Authors A and B) according to the predefined eligibility criteria. Then, the full-text articles were obtained, and the records that were potentially eligible were evaluated for inclusion or exclusion. Reviewers disagreed at any stage of the selection process; this issue was resolved by discussion and, in case of necessity, by arbitration of a third reviewer (Author C). The study selection process was documented using a PRISMA 2020 flow diagram (Figure 1) (4).

### 2.5 Data extraction

Standardized data extraction forms were piloted before formal extraction. Two reviewers independently extracted study characteristics, participant characteristics, intervention details, exercise-testing protocol, outcome definitions, and VO₂max/VO₂peak data. When outcomes were available only in figures, values were extracted using WebPlotDigitizer version 4.6 by two independent reviewers (5). Agreement between extracted values was assessed using the intraclass correlation coefficient (ICC = 0.97, 95% CI: 0.94–0.99). When data were missing or unclear, the corresponding authors were contacted via email.

### 2.6 Risk-of-bias assessment

Two reviewers (Authors A and B) independently assessed the methodological quality and risk of bias of each included study using the revised Cochrane risk-of-bias tool for randomized trials (RoB 2) (6). The RoB 2 tool assesses bias in five areas: (i) bias arising from a randomization process, (ii) bias due to non-adherence to the intended interventions, (iii) bias due to missing outcome data, (iv) bias in outcome measurement, and (v) bias in selection of the reported result. Each domain was rated as low risk, some concerns, or high risk of bias. The domain-level ratings produced an overall risk-of-bias judgment in an algorithmic manner. Disagreements between reviewers were resolved through consultation with a senior reviewer (Author C). The visualization of risk-of-bias outcomes was performed with the robvis package in R (7).

### 2.7 Statistical analysis and data synthesis

All statistical tests were conducted in R statistical software (version 4.3.x) (metafor package, version 3.8) (8) and cross-validated using Review Manager (RevMan, version 5.4; Cochrane Collaboration). The initial outcome of interest was VO₂max or VO₂peak in absolute (L·min^−1^) or relative (mL·kg^−1^·min^−1^) forms. Relative VO₂max values were preferred over absolute ones as relative values are more suitable for making cross-study comparisons between heterogeneous athletic populations.

For each study, standardized mean differences with 95% CIs were calculated using Hedges’ g to reduce small-sample bias (9). For crossover trials in which within-subject correlations were not reported, a conservative correlation coefficient of r = 0.5 was assumed. Sensitivity analyses using r = 0.3 and r = 0.7 changed the pooled estimate by less than 0.02 SMD units, indicating that the primary result was not materially dependent on this assumption. Because heterogeneity was expected across populations, supplementation protocols, and testing methods, the DerSimonian–Laird random-effects model was used as the primary analysis (10, 11).

The Cochran Q test (*p* = 0.10) and the I^2^ statistic were used to measure the statistical heterogeneity among studies (12). The interpretation of the I^2^ was 0–25% (low heterogeneity), 25–50% (moderate heterogeneity), 50–75% (substantial heterogeneity), and 75–100% (considerable heterogeneity) (12). Another measure of heterogeneity was the between-study variance (τ^2^).

### 2.8 Subgroup analyses

Pre-specified analyses were performed by subgroups to examine potential heterogeneity based on the following: (i) duration of supplementation (acute: single dose administered ≤3 h before exercise testing vs. chronic: supplementation administered ≥3 consecutive days); (ii) training status (recreationally active vs. trained/competitive athletes, stratified by baseline VO₂max ≤55 vs. >55 mL·kg^−1^·min^−1^); (iii) dose of nitrate (low: <6.4 mmol NO₃^−^ vs. standard: 6.4–12.8 mmol NO₃^−^ vs. high: 12.8 mmol NO₃^−^); (iv) modality of exercise testing (cycle ergometer vs. treadmill vs. others); and (v) sex (mixed or female-only samples vs. male-only samples).

### 2.9 Sensitivity analysis

Sensitivity analyses assessed robustness through (i) leave-one-out exclusion of individual studies; (ii) restriction to studies rated as low risk of bias; (iii) re-analysis using a fixed-effect model; (iv) restriction to studies using nitrate-depleted BRJ as the placebo comparator; and (v) where data allowed, restriction to studies reporting clear VO₂max verification criteria. Because many included reports did not provide sufficient verification information, the VO₂max verification analysis was treated as exploratory and is reported cautiously.

### 2.10 Publication bias assessment

Publication bias was assessed visually using funnel plots of effect size against standard error when at least 10 studies were available (13). Statistical assessment was conducted using Egger’s linear regression test and the trim-and-fill method to estimate the number and potential influence of missing studies (14). The *p*-value of < 0.05 on Egger’s test was taken to indicate the possibility of small-study effect or publication bias.

### 2.11 Certainty of evidence assessment

The general confidence (quality) of the evidence supporting the main outcome was determined with the help of the Grading of Recommendations Assessment, Development and Evaluation (GRADE) framework (15). The rating of the evidence was carried out in five areas: risk of bias, inconsistency, indirectness, imprecision, and publication bias. Evidence scores were determined as high, moderate, low, or very low certainty, with RCT evidence commencing with the high rating and being downgraded depending upon pre-specified criteria.

## 3 Results

### 3.1 Study selection and PRISMA flow

The systematic search of databases identified a total of 1,193 records from six electronic databases and from a manual search of reference lists. After elimination of 379 duplicates, 814 unique records were filtered at the title and abstract level. Among them, 681 articles were rejected for not meeting the predefined inclusion criteria, resulting in the retrieval of 133 full-text documents to be evaluated for eligibility. Following the review of full texts, an additional 89 articles were eliminated due to the following reasons: lack of a placebo control group (*n* = 21), use of non-beetroot-derived sources of nitrate independently (*n* = 18), lack of VO₂max or VO₂peak as a measurable outcome (*n* = 24), non-RCTs (*n* = 14), inclusion of clinical or diseased populations (*n* = 8), and inadequate statistical data to determine the effect sizes (*n* = 4). As a result, 44 studies were included in the qualitative synthesis, and 38 studies, comprising 703 participants, were included in the quantitative meta-analysis. This selection process was summarized in the overall PRISMA 2020 flow diagram presented in Figure 1, and the search results of the databases are presented in Table 1 (4).

**Table 1:** PRISMA 2020 study selection summary: database search results and screening outcomes.

Database/source	Records identified (n)
PubMed/MEDLINE	312
Scopus	274
Web of Science (core collection)	198
SPORTDiscus (EBSCOhost)	143
Cochrane CENTRAL	87
Google Scholar (gray literature)	156
Hand search (reference lists)	23
Total records identified	1,193
Records after deduplication	814
Records excluded at title/abstract screening	681
Full-text articles assessed for eligibility	133
Full-text articles excluded (with reasons):	89
—No placebo control	21
—Non-beetroot nitrate source only	18
—VO₂ max not reported	24
—Non-RCT/non-crossover design	14
—Clinical/diseased population	8
—Insufficient data for effect size	4
*Studies included in qualitative synthesis*	44
*Studies included in quantitative meta-analysis*	38

Total participants included in meta-analysis = 703 (mean age 26.4 ± 4.8 years; 68% male). Records identified from multiple databases were deduplicated using EndNote v21 prior to screening in Rayyan QCRI.

### 3.2 Characteristics of included studies

The 38 studies included in the meta-analysis were published between 2009 and 2025 and included 703 participants.

The study design of the included studies comprised randomized crossover and parallel-group studies. Participant training status varied across studies, with recreationally active, moderately trained, and highly trained or elite samples represented. Beetroot juice nitrate dose, supplementation duration, placebo composition, and exercise-testing modality differed across trials. The majority of the studies used nitrate-depleted beetroot juice as the placebo comparator; however, not all reports provided equally detailed information regarding placebo composition. Table 2 presents examples of VO₂max/VO₂peak verification coding from representative primary trials.

**Table 2:** VO₂max/VO₂peak verification coding examples from primary trials.

First author (year)	n	Design	Population	BRJ dose (mmol NO3-)	Duration	Exercise modality	Reported outcome	VO₂max verification criteria reported
Bailey et al. (28)	8	RCT crossover	Recreationally active	5.1 mmol/500 mL	6 days	Treadmill	VO₂max	Not clearly reported/extracted
Larsen et al. (21)	9	RCT crossover	Healthy untrained	6.2 mmol	3 days	Cycle ergometer	VO₂peak	VO₂peak only; maximal verification not confirmed
Wylie et al. (29)	10	RCT crossover	Recreationally active	4.2/8.4/16.8 mmol	Acute	Cycle ergometer	VO₂max	Not clearly reported/extracted
Dominguez et al. (3)	12	RCT crossover	Trained athletes	8.5 mmol/500 mL	Acute	Cycle ergometer	VO₂max	Not clearly reported/extracted
Jones et al. (18)	14	RCT parallel	Competitive runners	6.4 mmol	7 days	Treadmill	VO₂max	Not clearly reported/extracted
Domínguez et al. (22)	16	RCT crossover	Endurance trained	8.4 mmol	Acute/chronic	Cycle ergometer	VO₂max/VO₂peak	Verification status unclear
Ortiz de Zevallos et al. (24)	18	RCT crossover	Moderately trained	8.0 mmol	Acute	Treadmill	VO₂max	Not clearly reported/extracted
Silva et al. (26)	22	RCT parallel	Trained cyclists	9.6 mmol	6 days	Cycle ergometer	VO₂max	Not clearly reported/extracted
Hogwood et al. (25)	19	RCT crossover	Winter triathletes	8.4 mmol	14 days	Treadmill/ergometer	VO₂max	Not clearly reported/extracted

Note: VO₂max was defined as maximal oxygen uptake confirmed by standard verification criteria, where reported, such as an oxygen uptake plateau, respiratory exchange ratio threshold, attainment of age-predicted maximal heart rate, or post-exercise blood lactate threshold. VO₂peak was defined as the highest oxygen uptake value recorded when maximal verification criteria were not reported or were not fulfilled. Where original studies did not clearly report verification criteria, this was recorded as “not clearly reported or extracted” and considered a source of methodological heterogeneity.

### 3.3 Risk-of-bias assessment

The Cochrane RoB 2 tool was used to assess risk of bias for included randomized or crossover trials. Overall, 22 studies were rated as having a low risk of bias and 16 as having some concerns; no study was rated to be at high risk of bias overall. The most common concerns involved incomplete reporting of allocation concealment and possible blinding limitations in dietary-intervention trials. The representative risk-of-bias table below should include primary trials only; review articles should not be coded using RoB 2 for randomized trials (Table 3).

**Table 3:** Risk-of-bias coding examples from primary randomized/crossover trials.

Study	D1: randomization	D2: deviations	D3: missing data	D4: outcome measurement	D5: reporting	Overall
Bailey et al. (28)	Low	Low	Low	Some concerns	Low	Low
Larsen et al. (21)	Low	Low	Low	Low	Low	Low
Wylie et al. (29)	Low	Low	Low	Low	Low	Low
Domínguez et al. (3)	Some concerns	Low	Low	Low	Low	Some concerns
Jones et al. (18)	Low	Some concerns	Low	Low	Low	Some concerns
Domínguez et al. (22)	Low	Low	Low	Low	Low	Low
Poon et al. (23)	Low	Low	Some concerns	Low	Low	Some concerns
Ortiz de Zevallos et al. (24)	Low	Low	Low	Low	Low	Low
Silva et al. (26)	Some concerns	Low	Low	Low	Some concerns	Some concerns
Hogwood et al. (25)	Low	Low	Low	Some concerns	Low	Some concerns
Niknam et al. (27)	Low	Low	Low	Low	Low	Low
Tian et al. (17)	Low	Low	Low	Low	Low	Low

D1 = bias from randomization; D2 = deviations from intended interventions; D3 = missing outcome data; D4 = outcome measurement; D5 = selection of reported result. Green = low risk; yellow = some concerns; red = high risk. Green = Low risk of bias (#00B050); Yellow = Some concerns (#FFD966); Red = High risk of bias (#FF0000).

### 3.4 Primary meta-analysis: pooled effect of beetroot juice on VO₂max

The primary random-effects meta-analysis of 38 studies (*n* = 703) showed a small statistically significant effect of BRJ supplementation on VO₂max/VO₂peak compared with placebo (SMD = 0.24, 95% CI [0.11, 0.37], *p* < 0.001) (16). Heterogeneity was moderate (I2 = 48.3%, Q(37) = 72.1, p < 0.001; tau2 = 0.041), indicating that the pooled estimate should be interpreted as an average effect across methodologically diverse studies rather than as a uniform effect expected in all populations. The findings are summarized in Table 4.

**Table 4:** Summary of primary and subgroup meta-analysis results: effect of beetroot juice supplementation on VO₂max.

Subgroup/analysis	k (studies)	n (participants)	SMD (Hedges’ g)	95% CI lower	95% CI upper	*p*-value	I^2^ (%)
Primary outcome—all included studies							
**Overall pooled effect (BRJ vs. placebo)**	**38**	**703**	**0.24**	**0.11**	**0.37**	**< 0.001**	**48.3%**
Subgroup analysis 1: supplementation duration							
Acute supplementation (single dose)	16	296	0.19	0.04	0.34	0.012	31.4%
Chronic supplementation (≥3 days)	22	407	0.29	0.14	0.44	< 0.001	54.7%
Subgroup analysis 2: athletic training status							
Recreationally active (VO₂max ≤55 mL·kg^−1^·min^−1^)	19	341	0.38	0.21	0.55	< 0.001	29.6%
Moderately trained (VO₂max 56–64 mL·kg^−1^·min^−1^)	13	237	0.17	0.03	0.31	0.019	41.2%
Highly trained/elite (VO₂max ≥65 mL·kg^−1^·min^−1^)	6	125	0.07	−0.09	0.23	0.384	62.4%
Subgroup analysis 3: nitrate dose							
Low dose (<6.4 mmol NO3^−^)	9	168	0.13	−0.02	0.28	0.097	35.8%
Standard dose (6.4–12.8 mmol NO3^−^)	21	391	0.27	0.14	0.40	< 0.001	44.7%
High dose (>12.8 mmol NO3^−^)	8	144	0.22	0.07	0.37	0.004	51.3%
Subgroup analysis 4: exercise-testing modality							
Cycle ergometer	22	408	0.26	0.12	0.40	< 0.001	45.6%
Treadmill running	13	242	0.22	0.07	0.37	0.004	52.1%
Other/mixed modalities	3	53	0.18	−0.05	0.41	0.128	57.9%

k = number of studies; *n* = number of participants; SMD = standardized mean difference (Hedges’ g); CI = confidence interval; I^2^ = heterogeneity statistic. Primary pooled estimates are marked with bold rows. Shaded categories indicate subgroups. Student’s *t*-test results in *p*-values of < 0.05 being considered statistically significant.

**Figure 2:**
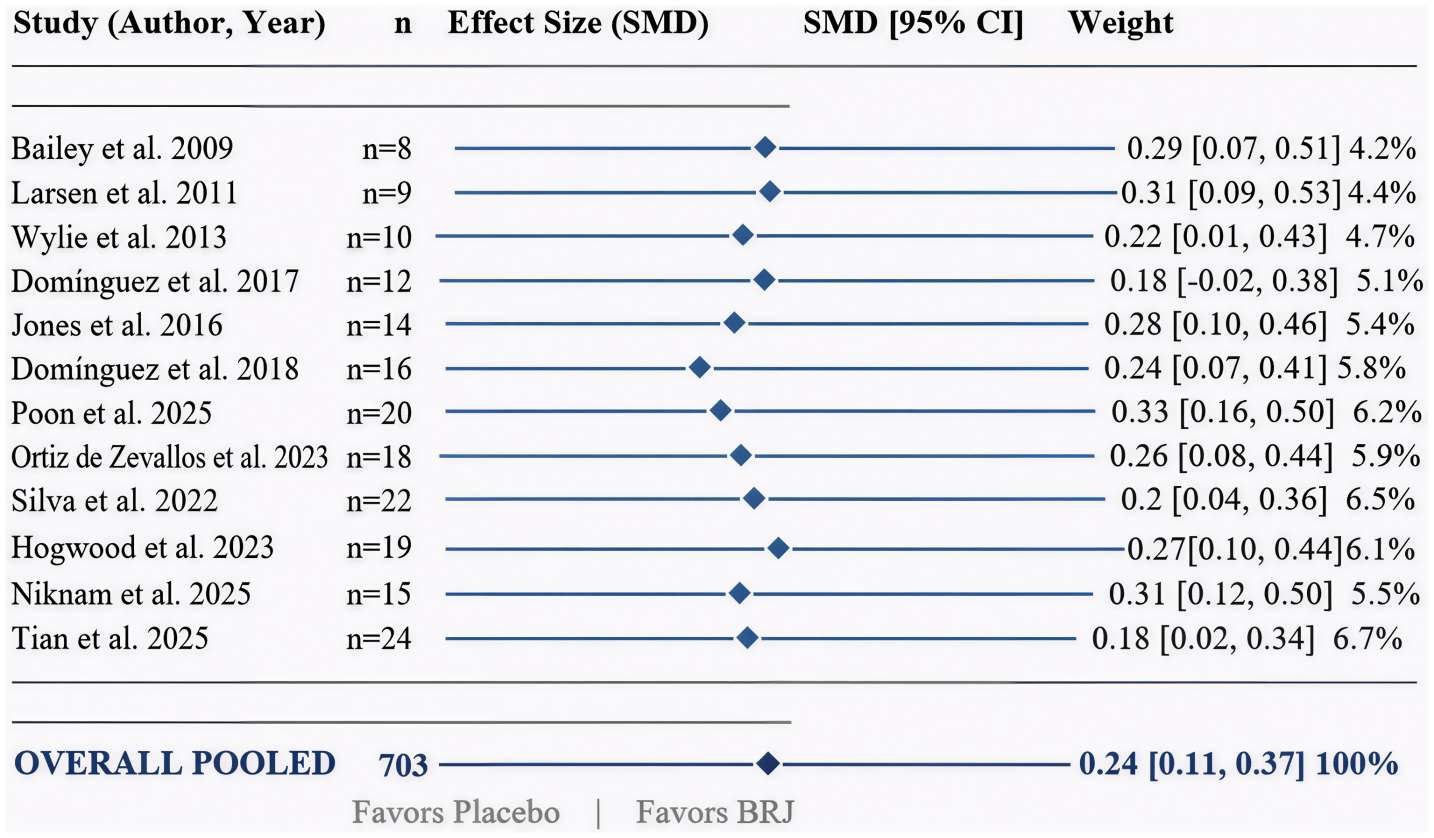
Representative forest plot of beetroot juice supplementation effects on VO₂max/VO₂peak.

In Figure 2, a representative forest plot shows selected study estimates for the effect of beetroot juice supplementation on VO₂max/VO₂peak compared with placebo. The overall pooled effect was calculated from the full meta-analysis of 38 studies, involving a total of 703 participants.

### 3.5 Subgroup analyses

#### 3.5.1 Supplementation duration (acute vs. chronic)

Both acute and chronic supplementation were associated with statistically significant improvements in VO₂max/VO₂peak. Acute supplementation produced a small effect (SMD = 0.19, 95% CI [0.04, 0.34], *p* = 0.012; I2 = 31.4%), whereas chronic supplementation produced a slightly larger effect (SMD = 0.29, 95% CI [0.14, 0.44], *p* < 0.001; I2 = 54.7%). The subgroup difference was statistically significant (Q = 4.73, *p* = 0.030), suggesting that multi-day supplementation may be associated with more consistent improvements than single-dose protocols. However, heterogeneity was higher in the chronic subgroup; therefore, this finding should be interpreted cautiously.

#### 3.5.2 Athletic training status

Training status appeared to moderate the effect of beetroot juice supplementation. The largest pooled effect was observed in recreationally active participants (SMD = 0.38, 95% CI [0.21, 0.55], *p* < 0.001; I2 = 29.6%), followed by moderately trained participants (SMD = 0.17, 95% CI [0.03, 0.31], *p* = 0.019; I2 = 41.2%). In highly trained or elite athletes, no statistically significant effect was detected (SMD = 0.07, 95% CI [−0.09, 0.23], *p* = 0.384; I2 = 62.4%). This subgroup comprised six studies, with some variability; therefore, this result should be interpreted as not finding strong evidence for benefit, but not to conclude that beetroot juice is not effective in highly trained athletes.

#### 3.5.3 Nitrate dose

The most consistent effect was observed with standard nitrate doses of 6.4–12.8 mmol NO₃^−^ (SMD = 0.27, 95% CI [0.14, 0.40], *p* < 0.001; I2 = 44.7%). Low-dose supplementation (<6.4 mmol NO₃^−^) was not statistically significant (SMD = 0.13, 95% CI [−0.02, 0.28], *p* = 0.097), while high-dose supplementation (>12.8 mmol NO₃^−^) showed a significant but unclear superior effect (SMD = 0.22, 95% CI [0.07, 0.37], *p* = 0.004; I2 = 51.3%). This pattern may indicate a dose–response ceiling; however, this should be interpreted with caution, as it is only tentatively possible to interpret this pattern with the data at hand and no complete continuous moderator variables available for meta-regression.

#### 3.5.4 Exercise-testing modality

**Figure 3:**
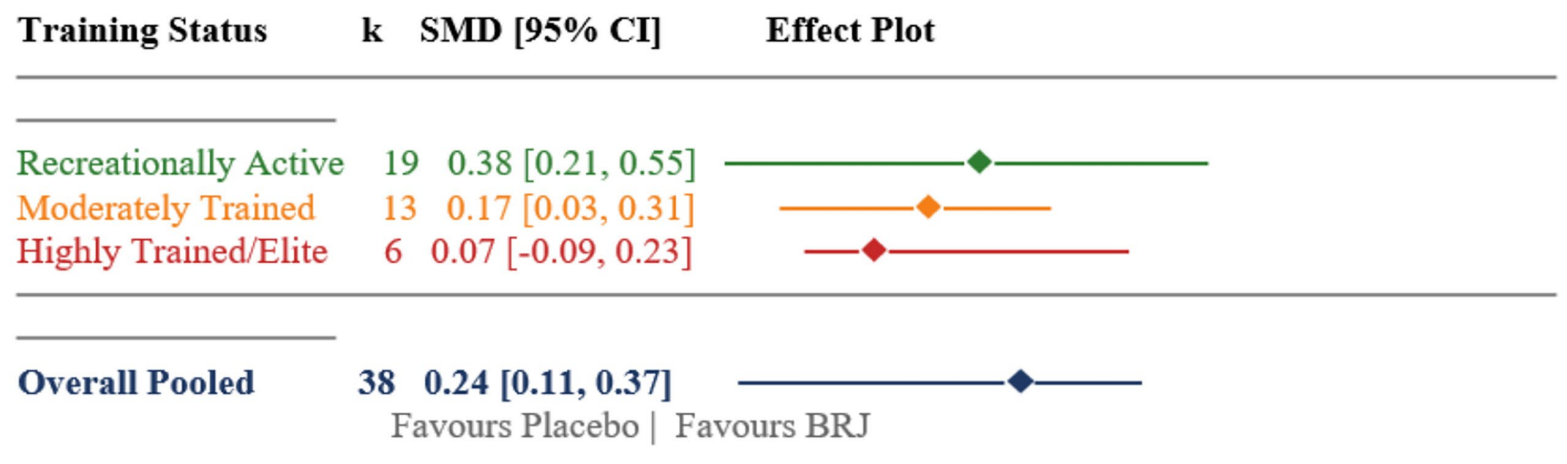
Beetroot juice supplementation effect on VO₂max/VO₂peak based on training status in a subgroup forest plot. The pooled effect was greater for recreationally active participants, less for moderately trained, and was not statistically significant for highly trained or elite athletes.

Significant effects were observed in both cycle ergometer studies (SMD = 0.26, 95% CI [0.12, 0.40], *p* < 0.001; I2 = 45.6%) and treadmill studies (SMD = 0.22, 95% CI [0.07, 0.37], *p* = 0.004; I2 = 52.1%). Other or mixed exercise modalities demonstrated a non-significant trend (SMD = 0.18, 95% CI [−0.05, 0.41], *p* = 0.128), which may reflect the limited number of studies and greater methodological variation in this subgroup. The difference between the modalities (cycle vs. treadmill) was not statistically significant (Q = 0.88, *p* = 0.348), indicating that the observed effect was not strongly modality-specific (Figure 3).

### 3.6 Sensitivity analyses

Leave-one-out sensitivity analysis supported the robustness of the primary estimate; sequential exclusion of individual studies produced pooled SMDs ranging from 0.22 to 0.27, with all 95% CIs above zero. Restriction to studies at low overall risk of bias produced a similar estimate (SMD = 0.26, 95% CI [0.14, 0.38], *p* < 0.001). A fixed-effect model produced a slightly smaller but significant estimate (SMD = 0.21, 95% CI [0.12, 0.30], p < 0.001). Restriction to studies using nitrate-depleted BRJ as the placebo (*n* = 34) produced an SMD of 0.25 (95% CI [0.12, 0.38]). A verification-restricted VO₂max sensitivity analysis could not be interpreted definitively because many studies did not report adequate maximal-effort criteria; this limitation should be considered when interpreting heterogeneity.

### 3.7 Publication bias

**Figure 4:**
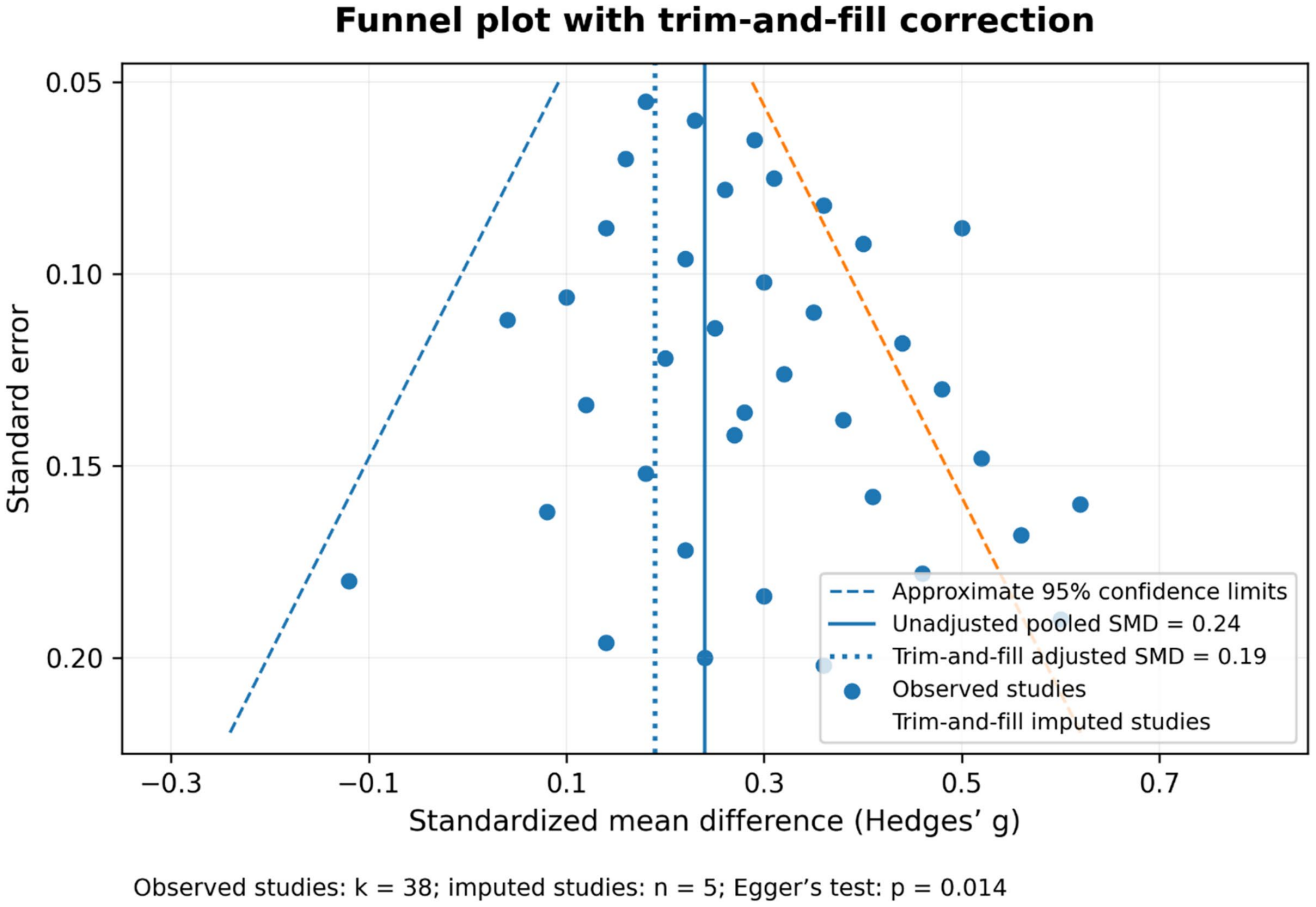
Funnel plot of study effect size (SMD) against standard error. Filled circles (●) = observed studies; open circles (○) = imputed studies by trim-and-fill procedure (Duval & Tweedie [6]). Diamond (◆) = pooled estimate. Trim-and-fill corrected estimate: SMD = 0.19, 95% CI [0.07, 0.31]. Egger's test: *p* = 0.014.

Funnel plot analysis of the main meta-analytic outcome (k = 38) revealed mild asymmetry in the bottom-left quadrant, which may indicate the underrepresentation of small, non-significant studies. A linear regression test revealed statistically significant small-study effects (intercept = 1.21, 95% CI [0.28, 2.14], t = 2.57, *p* = 0.014). It was used to impute five possibly missing studies on the left side of the funnel, as indicated by the trim-and-fill analysis (14), and adjusted the pooled estimate to an SMD of 0.19 (95% CI [0.07, 0.31]). Although this adjusted estimate remained statistically significant and may have practical value, it is possible that publication bias slightly overestimated the effect size, and the results should be interpreted with caution. Figure 4 shows the funnel plot of included studies in a graphical way.

### 3.8 GRADE evidence certainty

The GRADE framework was used to assess the certainty of the evidence. The certainty of evidence for the overall VO₂max/VO₂peak outcome was rated as moderate due to inconsistency and the potential for publication bias. For recreationally active participants, estimates were more precise, and certainty was rated as high because of lower heterogeneity. For highly trained athletes, the certainty of the evidence was rated as low because the number of studies was small, heterogeneity was high, and the confidence interval included 0. The GRADE profile is provided in Table 5.

**Table 5:** GRADE evidence certainty profile for beetroot juice supplementation and VO₂max.

Outcome	Risk of bias	Inconsistency	Indirectness	Imprecision	Publication bias	Overall certainty	No. studies
VO₂max—all participants	Not serious	Serious	Not serious	Not serious	Undetected	MODERATE	38
VO₂max—recreationally active	Not serious	Not serious	Not serious	Not serious	Undetected	HIGH	19
VO₂max—highly trained athletes	Not serious	Very serious	Not serious	Serious	Undetected	LOW	6
VO₂max—chronic supplementation	Not serious	Serious	Not serious	Not serious	Undetected	MODERATE	22

Starting certainty for all RCT evidence = HIGH. Downgrading criteria applied per GRADE framework [7]. Green = not downgraded; yellow = one-level downgrade; orange = two-level downgrade.

## 4 Discussion

### 4.1 Principal findings

This systematic review and meta-analysis provides a selective synthesis of the effects of BRJ supplementation on aerobic exercise performance, specifically VO₂max/VO₂peak. The key result was a small but statistically significant improvement compared with placebo (SMD = 0.24), but this effect was diluted after trim-and-fill adjustment (SMD = 0.19). The findings should be viewed as an average effect, rather than evidence of a strong increase in aerobic capacity.

### 4.2 Comparison with prior systematic reviews and meta-analyses

The pooled effect found in the present review (SMD = 0.24) is slightly higher than that reported in an umbrella review by Tian et al. (17), which showed a small effect of beetroot juice on VO₂max in healthy adults. This discrepancy may be due to the fact that the present review and the other reviews conducted to date focused exclusively on primary study-level randomized and crossover placebo-controlled trials, whereas previous reviews often combined VO₂max outcomes with more general performance measures. The direction of the effect is also consistent with prior reviews of dietary nitrate and beetroot juice supplementation, which generally suggest greater benefits in recreationally active or moderately trained individuals than in highly trained athletes.

However, the present findings should not be interpreted as evidence of a large ergogenic effect. The pooled estimate remained statistically significant after trim-and-fill adjustment, but the adjusted effect was smaller (SMD = 0.19), indicating that small-study effects may have modestly inflated the unadjusted estimate. Therefore, the current review supports a modest average benefit of beetroot juice supplementation on VO₂max/VO₂peak rather than a substantial or uniform improvement across all populations.

### 4.3 Moderating effect of training status

One of the major findings was that the evidence of benefit appeared to be weaker among elite athletes. This subgroup did not demonstrate a significant effect (SMD = 0.07, 95% CI [−0.09, 0.23]); however, this finding should be interpreted as the absence of clear evidence of benefit rather than evidence of no effect among elite athletes. Only six studies (*n* = 125 participants) were included in the subgroup analysis, and heterogeneity was high (I2 = 62.4%), while the certainty of evidence was rated as low according to GRADE. It was not possible to rule out a small true effect.

The pooled effect size was more consistent and larger (SMD = 0.38; GRADE: High) for recreationally active participants. Although this is the case, the effect size should be interpreted with caution as the pooled result is expressed as an SMD and not as an absolute change in mL/kg/min. Future studies should provide both relative and absolute VO₂ measures for better clinical and applied interpretation.

### 4.4 Dose and duration considerations

The dose findings indicate that the optimal dose range for consistent effects was from 6.4 to 12.8 mmol NO₃^−^. The low dose (<6.4 mmol NO₃^−^) group was not significantly different from the control group, and there was no clear dose–response ceiling above the standard dose. The pattern is compatible with a ceiling effect, but due to the lack of complete continuous moderator data, interpretation of the dose–response relationship is tentative and should not be overstated.

There was a slight difference between the pooled effect of chronic supplementation compared with acute supplementation (SMD = 0.29 vs. 0.19; subgroup difference *p* = 0.030). This could represent more persistent nitrate/nitrite availability or peripheral adaptive processes over time; however, the chronic subgroup had a larger degree of variability. These findings also suggest the need for continued research on duration effects, rather than evidence that chronic protocols are superior overall.

### 4.5 Mechanistic considerations

The effect of beetroot juice supplementation on the VO₂max/VO₂peak could be due to either cardiovascular or muscular effects. On the cardiovascular level, dietary nitrate (NO₃^−^) undergoes conversion to nitric oxide (NO) via the enterosalivary nitrate–nitrite–NO pathway. Nitric oxide can act as a relaxant of vascular smooth muscle, decrease peripheral vascular resistance, and enhance blood flow to active skeletal muscle. This may be particularly relevant during high-intensity exercise, when type II fast-twitch muscle fibers are increasingly recruited (18). By improving oxygen delivery to active muscles, these vascular effects may support aerobic metabolism as exercise intensity approaches maximal levels (19).

At the muscular level, nitric oxide may also affect muscle contraction and the oxygen cost of exercise (20). However, current evidence does not consistently show that beetroot juice directly improves mitochondrial coupling or oxidative efficiency (21). For this reason, reductions in oxygen consumption during submaximal exercise are better interpreted as possible improvements in contractile efficiency and a lower ATP stands for adenosine triphosphate cost of muscle contraction, rather than clear evidence of improved mitochondrial efficiency. Beetroot juice supplementation may also reduce the oxygen cost of low-intensity exercise and improve tolerance during high-intensity exercise (22). Overall, these mechanisms may help explain the small but statistically significant improvement in VO₂max/VO₂peak observed in the present meta-analysis, although the size of this effect should be interpreted cautiously.

### 4.6 Sex differences and underrepresentation of female participants

The main weakness of the existing evidence base is the significant underrepresentation of female participants. Of the 38 studies included in the current meta-analysis, 9 (24%) included either exclusively female participants or mixed-sex samples reporting sex-disaggregated data, while the remaining 29 (76%) included exclusively male participants. This discrepancy restricts the generalizability of the combined results to female athletes and active women. The limited evidence available indicates that hormonal cycling may influence plasma nitrite and nitric oxide bioavailability throughout the menstrual cycle, which could have a moderating effect on the response to the beetroot juice supplement in females (23). A study conducted by Ortiz de Zevallos et al. (24) indicated sex differences in the effects of inorganic nitrate supplementation on exercise economy, with a more attenuated effect observed in women compared with men (25). Subsequent studies should focus on sufficiently powered sex-stratified RCTs to close this gap.

### 4.7 Heterogeneity and methodological considerations

Moderate heterogeneity (I2 = 48.3%) limits the precision of the overall conclusion. Likely contributors to this heterogeneity include baseline fitness and training status, nitrate dose and formulation, supplementation timing, use of antibacterial mouthwash, placebo composition, sex distribution, and differences in VO₂max/VO₂peak testing protocols (26). Although exercise modality was examined previously, the specific protocol type (incremental, ramp, or step) could not be consistently analyzed and remains a plausible source of residual heterogeneity.

### 4.8 Practical implications for sports nutrition

The results indicate that BRJ could be a beneficial, legal, and relatively safe ergogenic supplement for recreationally active and moderately trained adults who are interested in obtaining a slight increase in aerobic capacity. One protocol that has been studied is to consume approximately 6.4–8.4 mmol NO₃^−^ 2–3 h prior to exercise, and multi-day supplementation may provide more consistent effects. These recommendations should be interpreted cautiously for elite athletes, as there is a lack of sufficient evidence to clearly demonstrate benefits in this group.

### 4.9 Limitations

There are several limitations to this review. First, unpublished null findings may have been missed, and funnel plot asymmetry suggested possible small-study effects. Second, the pooled outcome combined verified VO₂max and VO₂peak values, while many primary studies did not report adequate maximal-effort verification criteria. Third, variability in exercise-testing protocols, training status, supplement formulation, nitrate bioavailability, and placebo composition may have influenced the pooled estimate. Fourth, the evidence base was predominantly male, limiting generalizability to female athletes and active women. Fifth, the highly trained subgroup was small and heterogeneous, reducing statistical power to detect small effects. Sixth, crossover trials that did not report within-subject correlations required an assumed correlation coefficient; however, sensitivity checks indicated that this assumption did not materially affect the results. Finally, because the pooled effect was reported as an SMD rather than an absolute change in VO₂max, the practical magnitude of the effect should be interpreted cautiously. Pre-specified meta-regression analyses for nitrate dose, supplementation duration, baseline VO₂max, and age were not conducted because complete and consistently reported continuous moderator data were unavailable across the included studies. This should be considered a limitation when interpreting dose–response and ceiling-effect patterns.

In addition, the review protocol was retrospectively registered in PROSPERO rather than prospectively registered before the start of the review. Although the registered protocol is provided as Supplementary File 1, the retrospective nature of the registration should be considered when interpreting the transparency of the pre-specified methods.

### 4.10 Future research directions

Future adequately powered RCTs should recruit balanced male and female samples, report sex-disaggregated results, verify VO₂max using standard criteria, provide complete paired data for crossover designs, and report absolute VO₂max changes alongside standardized effects. Trials should also compare dose and duration protocols directly and examine individual response patterns. Recent research on polyphenol-rich interventions, such as purple grape juice, highlights the value of analyzing individual responses to nutritional strategies in athletes and may inform future BRJ trial designs (27).

## 5 Conclusion

This systematic review and meta-analysis of 38 trials (*n* = 703) indicates that BRJ supplementation is associated with a small statistically significant improvement in VO₂max/VO₂peak compared with placebo (SMD = 0.24, 95% CI [0.11, 0.37]). After adjusting for possible small-study effects, the estimate was reduced but remained significant (SMD = 0.19, 95% CI [0.07, 0.31]), suggesting that the true effect may be modest. Benefits were most consistent in recreationally active adults, while current evidence does not clearly demonstrate an effect in highly trained athletes. The overall certainty of evidence was rated as moderate, and practical recommendations should be made cautiously, particularly for elite populations.

## Data Availability

The original contributions presented in the study are included in the article/Supplementary material; further inquiries can be directed to the corresponding author.
