# Supplementary material for: Effects of beetroot juice supplementation on maximal oxygen uptake during aerobic exercise: a systematic review and meta-analysis
Source: Front Nutr. 2026 Jul 14;13:1858216. doi: 10.3389/fnut.2026.1858216 (PMC13407101; doi:10.3389/fnut.2026.1858216)
Supplement: Supplementary file 1 [file Data_Sheet_1.PDF]

NIHR | National Institute for Health and Care Research

PROSPERO
International prospective register of systematic reviews

Effects of Beetroot Juice Supplementation on Maximal or Peak Oxygen Uptake During Aerobic Exercise: A Systematic Review and Meta-analysis

Gang Qin, Sungmin Kim

Citation
Gang Qin, Sungmin Kim. Effects of Beetroot Juice Supplementation on Maximal or Peak Oxygen Uptake During Aerobic Exercise: A Systematic Review and Meta-analysis. PROSPERO 2026 CRD420261414721. Available from https://www.crd.york.ac.uk/PROSPERO/view/CRD420261414721.

REVIEW TITLE AND BASIC DETAILS

Review title

Effects of Beetroot Juice Supplementation on Maximal or Peak Oxygen Uptake During Aerobic Exercise: A Systematic Review and Meta-analysis

Condition or domain being studied

Aerobic Exercise; Competitive Athlete; Mineral Supplements; Nutrition Therapy

This review focuses on sports nutrition and exercise physiology, specifically beetroot juice or beetroot-derived dietary nitrate supplementation and its effects on maximal or peak oxygen uptake during aerobic exercise testing in healthy adults, recreationally active individuals, and trained or elite athletes.

Rationale for the review

Beetroot juice is a commonly used sports nutrition supplement because it is rich in inorganic nitrate, which may increase nitric oxide availability and influence oxygen delivery, mitochondrial efficiency, and exercise performance. Although previous reviews have examined dietary nitrate or beetroot juice supplementation in relation to endurance performance, maximal or peak oxygen uptake has often been treated as one of several secondary outcomes. A focused synthesis is needed to clarify whether beetroot juice supplementation improves VO_2max or VO_2peak, and whether the effect differs according to training status, supplementation duration, nitrate dose, and exercise testing modality.

Review objectives

The primary objective of this systematic review and meta-analysis is to evaluate the effect of beetroot juice or beetroot-derived dietary nitrate supplementation on maximal or peak

oxygen uptake, expressed as VO_2max or VO_2peak, during aerobic exercise testing in healthy adults. Secondary objectives are to examine whether the effect is moderated by training status, supplementation duration, nitrate dose, exercise testing modality, baseline aerobic fitness, age, and study design.

Keywords
Beetroot juice; Dietary nitrate; Nitrate supplementation; Nitric oxide

Country
Korea, Republic of

ELIGIBILITY CRITERIA

Population

Included
Healthy adults aged 18 years or older, including recreationally active individuals, trained athletes, and highly trained or elite athletes who completed aerobic exercise testing. Participants with diagnosed cardiovascular, pulmonary, metabolic, renal, or other clinical diseases that could directly affect aerobic capacity will be excluded.

Intervention(s) or exposure(s)

Included
Beetroot juice or beetroot-derived dietary nitrate supplementation, administered as an acute single dose or as repeated supplementation for three or more consecutive days. Eligible interventions may include nitrate-rich beetroot juice, beetroot concentrate, or beetroot-derived nitrate products with a reported nitrate dose. Studies using non-beetroot nitrate sources alone will be excluded.

Comparator(s) or control(s)

Included
PICO tags selected: Placebo

Eligible comparators will include placebo or control conditions, preferably nitrate-depleted beetroot juice or a taste-, color-, and volume-matched placebo. Studies will be included only if the comparator allows the independent effect of beetroot juice or beetroot-derived nitrate supplementation to be evaluated.

Excluded
Studies without a placebo or control condition will be excluded. Studies using another active nutritional supplement as the only comparator, or studies in which the independent effect of beetroot juice cannot be isolated, will also be excluded.

Study design
Only randomized study types will be included.

Included
Randomized controlled trials will be included, including parallel-group randomized controlled trials and randomized crossover trials evaluating beetroot juice or beetroot-derived dietary nitrate supplementation compared with placebo or control conditions.

Excluded

Non-randomized studies, observational studies, case reports, case series, reviews, editorials, conference abstracts without sufficient data, animal studies, and in vitro studies will be excluded.

Context

Studies conducted in laboratory, university, clinical research, or sport science settings will be eligible if maximal or peak oxygen uptake is assessed during incremental or graded aerobic exercise testing, including treadmill running, cycle ergometry, or other validated aerobic exercise modalities. No restrictions will be placed on country or publication year. Only full-text studies available in English will be included in the final analysis.

TIMELINE OF THE REVIEW

Date of first submission to PROSPERO

03 June 2026

Review timeline 1 change

Review start date: 1 January 2026. Review end date: 3 June 2026.

Date of registration in PROSPERO

03 June 2026

AVAILABILITY OF FULL PROTOCOL

Availability of full protocol

A full protocol has been written and uploaded to PROSPERO. The protocol may be accessed through this link https://www.crd.york.ac.uk/PROSPEROFILES/a04cdf6b95230cbe8bd7728ad4341819.pdf.

SEARCHING AND SCREENING

Search for unpublished studies

Both published and unpublished studies will be sought.

Main sources that will be searched

The main sources to be searched are *CENTRAL – Cochrane Central Register of Controlled Trials*, *Google Scholar*, *MEDLINE*, *SCIE – Science Citation Index Expanded* and *Scopus*.

Other sources that will be searched

SPORTDiscus via EBSCOhost; hand-searching of reference lists of included studies and relevant systematic reviews.

Search language restrictions

The review will only include studies published in English.

Search date restrictions

There are no search date restrictions.

Other methods of identifying studies

Other studies will be identified by: *reference list checking (backward citation searching).*

Additional information about identifying studies

Reference lists of included studies and relevant systematic reviews will be manually screened to identify additional eligible studies.

Link to search strategy

A full search strategy is available in the full protocol as described in the *Availability of full protocol* section

Selection process

Studies will be screened independently by at least two people (or person/machine combination) with a process to resolve differences.

Other relevant information about searching and screening

Search results will be deduplicated using EndNote and screened using Rayyan. The study selection process will be documented and reported using a PRISMA 2020 flow diagram.

DATA COLLECTION PROCESS

Data extraction from published articles and reports

Data will be extracted independently by at least two people (or person/machine combination) with a process to resolve differences.

Authors will be asked to provide any required data not available in published reports.

Study risk of bias or quality assessment

Risk of bias will be assessed using: *Cochrane RoB-2*

Data will be assessed independently by at least two people (or person/machine combination) with a process to resolve differences.

Additional information will be sought from study investigators if required information is unclear or unavailable in the study publications/reports.

Reporting bias assessment

Risk of bias due to missing results will be assessed using funnel plots when at least ten studies are available. Egger's regression test will assess funnel plot asymmetry, and the trim-and-fill method will be applied where appropriate.

Certainty assessment

Certainty of evidence will be assessed using the GRADE framework, considering risk of bias, inconsistency, indirectness, imprecision, and publication bias. Evidence certainty will be rated as high, moderate, low, or very low.

OUTCOMES TO BE ANALYSED

Main outcomes

The main outcome will be maximal or peak oxygen uptake measured during incremental or graded aerobic exercise testing. VO2max will refer to maximal oxygen uptake when maximal effort is verified according to criteria reported by the original study. VO2peak will

refer to the highest oxygen uptake value reported when maximal verification criteria are not clearly confirmed. Outcomes may be reported in relative units, such as mL/kg/min, or absolute units, such as L/min. When both are available, relative values will be preferred. The effect measure will be Hedges' g with 95% confidence intervals.

Additional outcomes

Additional outcomes will include subgroup and moderator–related outcomes used to interpret variation in the primary effect, including training status, supplementation duration, nitrate dose, exercise testing modality, baseline aerobic fitness, age, sex, and study design. These outcomes will be used for subgroup analyses or meta–regression where sufficient data are available.

PLANNED DATA SYNTHESIS

Strategy for data synthesis

A meta–analysis will be conducted where sufficient comparable data are available. The primary effect measure will be the standardized mean difference, calculated as Hedges' g with 95% confidence intervals. A random–effects model will be used because clinical and methodological heterogeneity is expected across studies. Heterogeneity will be assessed using Cochran's Q test, the I2 statistic, and between–study variance.

CURRENT REVIEW STAGE

Stage of the review at this submission

Review stage	Started	Completed
Pilot work	✓	✓
Formal searching/study identification	✓	✓
Screening search results against inclusion criteria	✓	✓
Data extraction or receipt of IPD	✓	✓
Risk of bias/quality assessment	✓	✓
Data synthesis	✓	✓

Review status

The review is completed.

Publication of review results

Results of the review will be published in English.

REVIEW AFFILIATION, FUNDING AND PEER REVIEW

Review team members

Dr Gang Qin (review guarantor and contact) ORCID: 0009–0000–4147–2870. hanyang. South Korea.

No conflict of interest declared.

Professor Sungmin Kim. hanyang. South Korea.

No conflict of interest declared.

Named contact

Dr Gang Qin (qingang@hanyang.ac.kr). ORCID: 0009-0000-4147-2870. hanyang. South Korea.

Review affiliation

Hanyang University

Funding source

Review has no funding and no agreed support from an academic institution and is done in authors' own time.

Additional information about funding

This review received no external funding.

Peer review

There has been no peer review of this planned review.

ADDITIONAL INFORMATION

Review conflict of interest

Declared individual interests are recorded under team member details.. No additional interests are recorded for this review.

Medical Subject Headings

Adult; Athletes; Dietary Supplements; Exercise; Exercise Test; Humans; Nitrates; Oxygen

Revision note 1 change

This revision was made to improve the accuracy and transparency of the registration record. The review timeline and current review stage were updated to reflect that the review has been completed, and the protocol details were revised to ensure consistency with the manuscript and submitted protocol. The record now clearly reflects retrospective registration.

SIMILAR REVIEWS

Check for similar records already in PROSPERO

PROSPERO identified a number of existing PROSPERO records that were similar to this one (last check made on 4 June 2026). These are shown below along with the reasons given by that the review team for the reviews being different and/or proceeding.

- Effects of Nitrate Supplementation on Female Exercise Performance: A Systematic Review and Meta Analysis [published 17 July 2025] [CRD420251106581]. The review was judged **not to be similar**
- A systematic review assessing the effects of nitrate supplementation on exercise economy, performance and recovery in menstruating or oral contraceptive pill using

healthy females [published 15 November 2023] [CRD42023474274]. The review was judged **not to be similar**

- Health Benefits of Beetroot supplementation in Older Adults: Cardiovascular, Cognitive, and Physical Exercise Insights: a systematic review. [published 6 December 2024] [CRD42024615605]. The review was judged **not to be similar**

PROSPERO version history 1 change

- Version 1.6, published 12 Jun 2026
- Version 1.5, published 03 Jun 2026
- Version 1.4, published 03 Jun 2026
- Version 1.3, published 03 Jun 2026
- Version 1.2, published 03 Jun 2026
- Version 1.1, published 03 Jun 2026
- Version 1.0, published 03 Jun 2026

Disclaimer

The content of this record displays the information provided by the review team. PROSPERO does not peer review registration records or endorse their content.

PROSPERO accepts and posts the information provided in good faith; responsibility for record content rests with the review team. The guarantor for this record has affirmed that the information provided is truthful and that they understand that deliberate provision of inaccurate information may be construed as scientific misconduct.

PROSPERO does not accept any liability for the content provided in this record or for its use. Readers use the information provided in this record at their own risk.

Any enquiries about the record should be referred to the named review contact
