# Supplementary material for: Effects of beetroot juice supplementation on maximal oxygen uptake during aerobic exercise: a systematic review and meta-analysis
Source: Front Nutr. 2026 Jul 14;13:1858216. doi: 10.3389/fnut.2026.1858216 (PMC13407101; doi:10.3389/fnut.2026.1858216)
Supplement: Supplementary file 2 [file Data_Sheet_2.PDF]

Completed PRISMA 2020 Checklist

Paper analysed: Effects of Beetroot Juice Supplementation on Maximal Oxygen Uptake During Aerobic Exercise: A Systematic Review and Meta-Analysis

Critical compliance summary: The paper reports most core PRISMA items, but it is weak on complete database-specific search strategies, individual excluded-study citations, full included-study characteristics, full study-level risk-of-bias/effect-size tables, protocol amendments, funding, competing interests, and data/code availability.

Reported	Partly reported	Not reported	Total checklist items
31	7	4	42

Section	Topic	Item #	Checklist item	Assessment	Location where item is reported / notes
TITLE	Title	1	Identify the report as a systematic review.	Reported	Title page / p.1: title states "Systematic Review and Meta-Analysis".
ABSTRACT	Abstract	2	See the PRISMA 2020 for Abstracts checklist.	Reported	Abstract / p.1: structured Background, Methods, Results, Conclusion, Keywords.
INTRODUCTION	Rationale	3	Describe the rationale for the review in the context of existing knowledge.	Reported	Introduction / p.1: explains VO2max/VO2peak relevance, nitrate-NO pathway, and gaps in prior reviews.
INTRODUCTION	Objectives	4	Provide an explicit statement of the objective(s) or question(s) the review addresses.	Reported	Introduction / p.1: states aim to include RCTs on beetroot juice effects on VO2max/VO2peak and explore moderators.
METHODS	Eligibility criteria	5	Specify inclusion/exclusion criteria and how studies were grouped for syntheses.	Reported	Methods 2.2 / p.2: RCT/crossover, BRJ/nitrate, placebo, VO2max/VO2peak; exclusions listed. Grouping also in 2.8 / pp.4-5.
METHODS	Information sources	6	Specify databases/registers/websites/other sources and date last searched.	Reported	Methods 2.3 / p.2: PubMed/MEDLINE, Scopus, Web of Science, SPORTDiscus, CENTRAL, Google Scholar; searched to 31 Jan 2026; hand-searching noted.
METHODS	Search strategy	7	Present full search strategies, including filters and limits.	Partly reported	Methods 2.3 / p.2: Boolean query is provided, but exact database-specific strategies, filters, and limits are not fully reproduced for every database.
METHODS	Selection process	8	Specify screening methods, reviewer numbers, independence, disagreements, automation tools.	Reported	Methods 2.4 / p.2 and Figure 1 / p.3: EndNote v21, Rayyan QCRI, two independent reviewers, third reviewer arbitration.
METHODS	Data collection process	9	Specify data collection methods, reviewer numbers, independence, contacting investigators, automation tools.	Reported	Methods 2.5 / p.4: piloted extraction forms, two independent reviewers, WebPlotDigitizer for figures, ICC, author contact for missing/unclear data.
METHODS	Data items	10a	List and define all outcomes for which data were sought.	Reported	Methods 2.1 / p.2 and 2.7 / p.4: VO2max/VO2peak defined; relative and absolute oxygen uptake described.
METHODS	Data items	10b	List and define other variables sought and assumptions about missing/unclear information.	Reported	Methods 2.5 / p.4: study, participant, intervention, testing, and outcome data; Methods 2.7 / p.4 includes assumed crossover correlation r=0.5.
METHODS	Study risk of bias assessment	11	Specify risk-of-bias methods, tools, reviewers, independence, automation tools.	Reported	Methods 2.6 / p.4: Cochrane RoB 2, two independent reviewers, senior reviewer resolution, robvis package.
METHODS	Effect measures	12	Specify effect measures used.	Reported	Methods 2.7 / p.4: standardized mean differences using Hedges g with 95%

Section	Topic	Item #	Checklist item	Assessment	Location where item is reported / notes
					CIs.
METHODS	Synthesis methods	13a	Describe processes to decide which studies were eligible for each synthesis.	Reported	Methods 2.2 / p.2, 2.7 / p.4, 2.8 / pp.4-5: eligibility and subgroup/synthesis categories specified.
METHODS	Synthesis methods	13b	Describe data preparation methods.	Reported	Methods 2.5 / p.4 and 2.7 / p.4: WebPlotDigitizer, relative VO2 preference, Hedges g, crossover correlation assumption and sensitivity checks.
METHODS	Synthesis methods	13c	Describe tabulation/visual display methods.	Reported	Results tables and figures: Table 1 / pp.5-6, Table 4 / p.8, Figures 1-4 / pp.3, 9-11.
METHODS	Synthesis methods	13d	Describe synthesis methods and rationale; meta-analysis model, heterogeneity, software.	Reported	Methods 2.7 / p.4: R 4.3.x/metafor, RevMan, DerSimonian-Laird random effects, Q, I2, tau2.
METHODS	Synthesis methods	13e	Describe methods to explore heterogeneity.	Reported	Methods 2.8 / pp.4-5: pre-specified subgroup analyses by duration, training status, dose, modality, sex.
METHODS	Synthesis methods	13f	Describe sensitivity analyses.	Reported	Methods 2.9 / p.5: leave-one-out, low-risk-only, fixed-effect model, nitrate-depleted placebo, VO2max verification restrictions.
METHODS	Reporting bias assessment	14	Describe methods to assess risk of bias due to missing results.	Reported	Methods 2.10 / p.5: funnel plot, Egger regression, trim-and-fill.
METHODS	Certainty assessment	15	Describe methods to assess certainty/confidence.	Reported	Methods 2.11 / p.5: GRADE domains and rating levels.
RESULTS	Study selection	16a	Describe search and selection results, ideally using a flow diagram.	Reported	Figure 1 / p.3 and Results 3.1 / pp.5-6: 1,193 records, 379 duplicates, 814 screened, 44 qualitative, 38 quantitative.
RESULTS	Study selection	16b	Cite studies that appeared eligible but were excluded and explain why.	Partly reported	Results 3.1 / pp.5-6 gives exclusion reasons and counts, but does not cite each excluded near-eligible study individually.
RESULTS	Study characteristics	17	Cite each included study and present its characteristics.	Partly reported	Results 3.2 / pp.6-7 and Table 2 provide representative trial characteristics, but not a complete characteristics table for every included study.
RESULTS	Risk of bias in studies	18	Present risk-of-bias assessments for each included study.	Partly reported	Results 3.3 / pp.7-8 reports overall counts and a representative RoB table, but not full RoB judgments for all included studies.
RESULTS	Results of individual studies	19	Present summary statistics/effect estimates and precision for each study.	Partly reported	Figure 2 / p.9 shows representative study estimates; full individual-study statistics for all 38 studies are not shown.
RESULTS	Results of syntheses	20a	Summarise characteristics and risk of bias among contributing studies.	Partly reported	Results 3.2-3.4 / pp.6-8 provide characteristics and RoB overview; synthesis-specific RoB details are limited.
RESULTS	Results of syntheses	20b	Present results of all statistical syntheses, summary estimates, precision, heterogeneity.	Reported	Results 3.4 and Table 4 / p.8; subgroup results / pp.9-10; sensitivity / p.10.
RESULTS	Results of syntheses	20c	Present investigations of possible causes of heterogeneity.	Reported	Results 3.5 / pp.9-10: duration, training status, dose, modality subgroup analyses.
RESULTS	Results of syntheses	20d	Present sensitivity analyses.	Reported	Results 3.6 / p.10: leave-one-out, low-risk-only, fixed-effect, nitrate-depleted placebo, VO2 verification.
RESULTS	Reporting biases	21	Present assessments of risk of bias due to missing results.	Reported	Results 3.7 / pp.10-11 and Figure 4 / p.11: funnel asymmetry, Egger p=0.014, trim-and-fill adjusted estimate.

Section	Topic	Item #	Checklist item	Assessment	Location where item is reported / notes
RESULTS	Certainty of evidence	22	Present certainty assessments for each outcome.	Reported	Results 3.8 and Table 5 / p.11: GRADE profile for overall, recreationally active, highly trained, chronic supplementation.
DISCUSSION	Discussion	23a	Provide general interpretation in context of other evidence.	Reported	Discussion 4.1-4.2 / p.12: modest effect, comparison with prior reviews.
DISCUSSION	Discussion	23b	Discuss limitations of the evidence included.	Reported	Discussion 4.6-4.9 / pp.13-14: female underrepresentation, heterogeneity, small elite subgroup, publication bias.
DISCUSSION	Discussion	23c	Discuss limitations of review processes used.	Reported	Discussion 4.9 / pp.13-14: no registration, combined VO2max/VO2peak, assumed crossover correlations, no meta-regression.
DISCUSSION	Discussion	23d	Discuss implications for practice, policy, and future research.	Reported	Discussion 4.8 / p.13 and 4.10 / p.14: practical sports nutrition guidance and future RCT recommendations.
OTHER INFORMATION	Registration and protocol	24a	Provide registration information or state the review was not registered.	Reported	Methods 2.1 / p.2: protocol was not prospectively registered in PROSPERO.
OTHER INFORMATION	Registration and protocol	24b	Indicate where protocol can be accessed, or state that no protocol was prepared.	Partly reported	Methods 2.1 / p.2 states no prospective PROSPERO registration, but does not clearly say where a protocol can be accessed or that none was prepared.
OTHER INFORMATION	Registration and protocol	24c	Describe and explain amendments to registration/protocol information.	Not reported	No amendments statement located.
OTHER INFORMATION	Support	25	Describe financial/non-financial support and role of funders/sponsors.	Not reported	No funding/support statement located.
OTHER INFORMATION	Competing interests	26	Declare competing interests of review authors.	Not reported	No competing-interest declaration located.
OTHER INFORMATION	Availability of data, code and materials	27	Report public availability of forms, extracted data, analytic code, and materials.	Not reported	No data/code/material availability statement located.

Required fixes before submission

- Add an appendix with full database-specific search strategies for PubMed, Scopus, Web of Science, SPORTDiscus, CENTRAL, and Google Scholar, including exact filters/limits.
- Add a table listing key full-text exclusions with citations, not only counts by reason.
- Add complete characteristics for every included study, not only representative examples.
- Add full RoB 2 domain ratings for every included study, or attach them as supplementary material.
- Add a complete individual-study results table with effect sizes, 95% CIs, and weights for all 38 studies.
- Clarify protocol status: either provide protocol access details or state explicitly that no protocol was prepared; add an amendments statement.
- Add funding/support, competing interests, and data/code/material availability statements even if the answer is "none" or "available on request".

Prepared from the uploaded PRISMA 2020 checklist and the uploaded manuscript. Page numbers refer to the manuscript pagination visible in the uploaded file.
